# Factors associated with irritable bowel syndrome among medical students at Ain Shams University

**DOI:** 10.1186/s42506-019-0023-8

**Published:** 2019-12-04

**Authors:** Doaa Elhosseiny, Nehal Elfawy Mahmoud, Ayat F. Manzour

**Affiliations:** 1 Department of Community, Environmental & Occupational Medicine, Faculty of Medicine, Ain Shams University, Cairo, Egypt; 2 Department of Internal Medicine, Faculty of Medicine, Ain Shams University, Abbasia Square, Cairo, Egypt

**Keywords:** Irritable bowel syndrome, Medical students, Hospital Anxiety and Depression Scale, Stress-related diseases, Anxiety

## Abstract

**Background:**

Irritable bowel syndrome (IBS) is one of the most common and potentially disabling gastrointestinal disorders. The pathogenesis of this disorder remains obscure. However, many etiological explanations point toward bacterial etiology. Also, several studies have documented that psychological and social factors may play a role. Medical education is among the most challenging and stressful education, and this may predispose to high rates of IBS.

**Objectives:**

The aims of this study are to estimate the frequency of IBS in a selected sample of students of Faculty of Medicine in Ain Shams University and to find out the determinants associated with this disorder.

**Study design:**

A cross-sectional study was carried out among medical students from October 2017 to February 2018 at Faculty of Medicine—Ain Shams University. All participants were asked to complete a confidential self-administered questionnaire. An interview questionnaire was used for diagnosis of IBS according to Rome III criteria, while morbid anxiety and depression were diagnosed by using the Arabic version of Hospital Anxiety and Depression Scale (HADS).

**Results:**

Three hundred eighty-two students completed the questionnaire. The frequency of IBS was 31.7% with higher proportion among females and among students with positive family history of IBS. However, IBS was significantly less prevalent among students practicing regular exercise. Lastly, based on (HADS), there was a statistical significant relationship between IBS and anxiety (*p* < 0.05), but not depression.

**Conclusion:**

The study revealed that around 31% of the studied group was suffering from IBS. Female gender, suffering from anxiety, and positive family history of IBS were the main associated factors for IBS. Screening of all medical students in the faculty for IBS is suggested. Providing psychological and emotional support along with stress management is highly recommended.

## Introduction

Irritable bowel syndrome (IBS) is one of the most common and potentially disabling gastrointestinal disorders characterized by pain in abdomen, bloating, and alteration in a person’s bowel habits, but without any organic pathology [1]. Traditionally, in practical work, IBS was diagnosed by exclusion, but recently, Rome criteria was used as a golden tool for diagnosis of IBS in researches and clinical work. According to Rome III, three types of IBS have been recognized; (1) diarrhea-predominant, (2) constipation-predominant, and (3) alternating diarrhea and constipation [2]. The prevalence of IBS varies from 5.7 to 34% worldwide, with a wide variation based on the tool used [3]. According to Rome III criteria, Western population have higher prevalence of IBS (ranges from 10 to 15%) than Asian ones (ranges from 1 to 10%) [4]. Arab countries are among the least studied populations in the world. A recent meta-analysis of the global prevalence of IBS revealed no studies done from any Arab country [3]. However, limited data are available on IBS in some countries, including an Egyptian study conducted in an urban area in Suez governorate which revealed high prevalence rate of 34.2% among the studied group [5]. Another study conducted in Saudi Arabia showed a prevalence of 11.4% [6].

The burden of this disorder on healthcare systems is major. In the USA, it was reported that 8 billion dollars were spent annually on medical cost of IBS patients. In addition, many patients underwent unnecessary surgeries such as appendectomy or hysterectomy due to difficulty in diagnosis in some atypical cases [7].

Also, the quality of life for patients suffering from IBS is greatly affected physically, psychologically, and economically. Several cases reported difficulties in concentration, decreased energy level, and lower self-esteem [8]. IBS has also induced an occupational hazard as it affects the performance of patients at their jobs. An interesting Iranian study reported that IBS was the second leading cause of lost work days after common cold [9]. In addition, many cases become socially embarrassed due to unpredictable bowel habits [10].

Although half of the cases referred to gastroenterologists is due to IBS, no clear etiology was found; however, some researches revealed that certain factors such as psychological factors, dietary habits, and exercise level were related to IBS onset and course [10]. There might be also a genetic role in the etiology of IBS as 33% of patients reported a positive family history [11]. Moreover, personal factors including age and gender might influence the occurrence of IBS. It has been reported that females are more vulnerable than males and those in their late teens and twenties have the highest risk [11].

Anxiety and depression can affect many university students particularly those studying medicine. They are under constant stress due to the long duration of their study with high work overload and mental exhaustion due to numerous exams [12, 13]. A recent Chinese study compared the prevalence of IBS among students in different universities and found that medical students had the highest level of IBS compared to engineering and science students [14]. In Saudi Arabia, a study conducted on medical students and interns in Jeddah reported a prevalence of IBS of 31.8% [15]. In Egypt, information on the prevalence of IBS and its associated factors among university students is deficient; a recent study reported 22.9% prevalence rate among Suez Canal University students [16]. To our knowledge, there is a lack of studies regarding the epidemiology of IBS among Egyptian medical students.

The aims of this study are as follows:

1. To measure the frequency of IBS and its subtypes in a sample of medical students based on Rome III criteria.
2. To identify associated factors related to IBS (socio-demographic, some life-style habits, dietary habits).
3. To investigate the relation between IBS and emotional disturbance as regards morbid anxiety and depression among the study group.

## Participants and methods

### Study design

Analytical cross-sectional study was conducted in 4 months duration from October 2017 to February 2018.

### Study setting

This study was carried out at the Faculty of Medicine, Ain Shams University, located in El-Abassyia, Cairo, Egypt.

### Participants and sample size

Medical students studying at the Faculty of Medicine in the academic year (2017–2018) from the first to the sixth educational years with their age ranges from 18 to 25 years were included in the study. Participants with known organic gastrointestinal disorders or individuals with alarming symptoms like marked weight loss and bloody stools were excluded.

#### Study sample

Sample size was calculated using Open-epi; an open source software for epidemiological statistics, assuming hypothesized percentage of IBS in the population = 31.8% on the basis of a previous study [15] and 95% confidence limits. The minimum calculated sample size to achieve study objectives was 334. Sample size was increased to 400 students to compensate for non-response. Proportionate stratified random sampling was used to allocate participants based on percentage of students in each academic year. Response rate was 95.5% (382). Reasons for non-participation were lack of time and refusals.

### Tools of the study

A validated pre-constructed anonymous questionnaire was used. The questionnaire composed of four parts:

#### First tool

A self-administered questionnaire was used to collect baseline information such as socio-demographic and academic data; family history of IBS; previous diagnosis of IBS by a physician; other chronic medical conditions; daily life habits such as number of sleeping hours, smoking, exercise, and so on; Traveler’s diarrhea and whether its presence triggered the first onset of IBS; and negative life events during childhood such as death of a close family member, parents’ divorce, or experiencing serious illness. Regular exercise was defined as practicing any type of sport for 30 min three times per week. Those who smoked greater than 100 cigarettes in their lifetime and has smoked in the last 28 days were considered as current smokers. Enough income was defined as income more than 3600 Egyptian pounds [17]. Travelers’ diarrhea was defined as suffering from abrupt onset of diarrhea that might be associated with fever, bloating, and loss of appetite during the trip.

#### Second tool

Food Frequency Questionnaire (FFQ) was self-administered and was used for dietary assessment inquiring about frequency of use of different food items such as milk, yogurt, fruit and vegetables, fish, fast food, homemade food, and so on. The FFQ is widely used for dietary assessment in epidemiologic research. The collected data was used to calculate nutrient intake and to relate consumption of foods to specific disease outcomes [18].

#### Third tool

*The English version of Rome III questionnaire* was used for IBS diagnosis [2] The Rome III criteria is a standard tool used widely in the diagnosis of functional gastrointestinal disorders. Rome III Criteria can rationally diagnose IBS in the absence of red flag symptoms. The sensitivity of Rome III Criteria is 65%, specificity is 100%, and the positive predictive value is 100% [2, 19]. Students were interviewed by an expert for accurate diagnosis of cases. IBS was defined according to Rome III criteria as recurrent abdominal pain for at least 3 days per month during the past 3 months, associated with two or more of the following features: (1) improvement with defecation; and/or (2) onset associated with a change in stool frequency; and/or (3) onset associated with a change in stool appearance. Individuals with IBS were divided into (1) diarrhea-predominant, (2) constipation-predominant, and (3) alternating diarrhea and constipation according to the proportion of lumpy and hard stools. Reliability was assessed with Cronbach’s α by the researches and was found to be 0.75.

#### Fourth tool

The Arabic version of Hospital Anxiety and Depression Scale (HADS) [20] was self-administered and was used to identify individuals with clinically significant symptoms of anxiety and depression. It is a standardized, valid, and reliable self-report rating scale. It has 14 questions; seven questions for anxiety and seven for depression, for the answers, four-point Likert scale is used ranging from 0 (not present) to 3 (considerable). In addition, anxiety and depression scores are summed separately. For each subscale, the scores can be divided into 0–7 (normal), 8–10 (borderline), and over 11 (cases). Reliability was assessed with Cronbach’s α and was found to be 0.82.

A pilot study was conducted prior to data collection on ten participants to test clarity and applicability of the questionnaires, and necessary modifications were done in some questions.

### Ethical consideration

Ethical approval was obtained from ethical committee, Faculty of Medicine, Ain Shams University. An informed written consent was obtained from all the students after informing them about the aim of the study and assuring the confidentiality of all provided data and that no personal identifying data would be used in the study.

### Data management and statistical analysis

All eligible questionnaires were coded. Data analysis was performed using the SPSS 20.0 (SPSS Inc., Chicago, IL). Distributions of sex and lifestyle factors were analyzed by Pearson’s χ^2^. BMI = kg/m^2^ where kg is a person’s weight in kilograms and m^2^ is their height in meters squared. BMI was categorized as follows: underweight (< 18.5), normal weight (18.5–24.9), overweight (25–29.9), while obesity is considered when BMI is 30 or greater. Student *t* test was used to compare the anxiety and depression levels between groups. Quantitative data were presented as mean ± SD. All calculated *p* values were two-tailed and *p* < 0.05 was considered statistically significant. Multivariate logistic regression was utilized to find out factors associated with IBS.

## Results

This cross-sectional study included 400 medical students; 382 completed the questionnaire. Rome III criteria questionnaire was used to diagnose IBS. It was found that 121 students were positive giving an IBS frequency of 31.7% among studied medical students. Examining subtypes of IBS revealed that 26.6% were diarrhea predominant (IBS-D) while 73.4% were constipation predominant (IBS-C).

The Majority of studied sample were males (66.5%), 53.1% were in junior years (1st, 2nd, and 3rd years), while 46.9% were in senior years. Their mean age was 20.69 ± 3.99. Only 4.5% of study sample's grade point average (GPA) was excellent while 32.5% had accepted and 45.8% had good/very good assessment in previous years. Seven percent only were married while the rest were single. Almost half (54.8%) their parents had enough monthly income or even exceeds their needs. Overweight/obese students comprised 31.9, 1.3% were underweight, while 65.8 were within normal BMI. Further, 8.5% were smokers, 37% performed regular exercise,53.3% used to drink eight water cups or more daily, only 24.5% used to have breakfast every day while half (50.2%) used to drink fluids along with having their meals. Analyzing HADS indicated that 32.9% have morbid anxiety, 26.1% had depression, and 26.3% were borderline for anxiety while 27.2% were borderline for depression. Regarding family history of IBS, 35.8% of students had positive family history while 14.3% have different chronic diseases. Traveler’s diarrhea was reported in 8%, food allergy in 14.8%, while 16.8% were previously diagnosed as IBS patients. Almost two-thirds of participants (68.5%) had sources of emotional stress in the past 6 months. Food frequency questionnaire analysis revealed that almost two-thirds of students eat vegetables (66.8%), fruits (66.9%), milk, and dairy products as well as eggs (64%) and home-made food (69.8%) three or more times weekly. Most of participants eat fish (89.2%) and nuts (87.6%) twice or less weekly. High-fat diet and fast food were eaten three times or more weekly in 30.8% and 39.7% respectively. Regarding caffeine drinking, 52.6% used to drink coffee and 63.6% used to drink tea three or more times every week. Around half of studied sample eat fried food and processed food on weekly basis (three times or more) (49% and 45.6% respectively). One-third used to eat spicy food frequently (three times or more) (35%) while most of them use artificial sweeteners twice/week or less.

Table 1 shows that there was a statistically significant differences between IBS students and free students regarding sex (*p* = 0.006), academic stage (*p* = 0.04), and family history of IBS (*p* = 0.012). No statistically significant differences regarding other socio-demographic characteristics were detected.

**Table 1:** Comparison between IBS patients and normal students regarding socio-demographic characteristics

	Normal *N* (%)	IBS patient *N* (%)	Chi-square	*p*
Sex				
Male	183 (70.1)	67 (55.4)	7.95	0.006^a^
Female	78 (29.9)	54 (44.6)		
GPA				
Accepted	92 (41.1)	34 (38.2)	0.624	0.892
Good	77 (34.4)	30 (33.7)		
Very good	44 (19.6)	19 (21.3)		
Excellent	11(4.9)	6 (6.7)		
Academic stage				
Junior years	148 (56.7)	55 (45.5)	4.202	0.04^a^
Senior years	113 (43.3)	66 (54.5)		
Living condition				
With family	208 (79.7)	90 (74.4)	1.361	0.243
Private house	53 (20.3)	31 (25.6)		
Income				
Enough and exceeds	150 (57.5)	63 (52.1)	0.979	0.322
Enough only or not enough	111 (42.5)	58 (47.9)		
Parents				
Living together	230 (89.8)	100 (83.3)	3.225	0.073
Divorce/dead (one or both)	26 (10.2)	20 (16.7)		
BMI				
Underweight	3 (1.3)	6 (5.1)	4.508	0.105
Normal	143 (62.4)	73 (61.9)		
Overweight	55 (24.0)	32 (27.1)		
Obese	28 (12.2)	7 (5.9)		
Family History of IBS				
Yes	57 (21.8)	80 (66.1)	6.289	0.012^a^
No	204 (78.2)	41 (33.9)		

^a^Significant

Table 2 shows that there was a statistically significant difference regarding regular exercise performance between IBS students and normal students (*p* = 0.007). No statistically significant differences regarding other lifestyle characteristics were detected.

**Table 2:** Comparison between IBS patients and normal students regarding lifestyle characteristics

	Normal *N* (%)	IBS patient *N* (%)	Chi-square	*p*
Regular exercise				
Yes	111 (42.5)	34 (28.1)	7.309	0.007^a^
No	150 (57.5)	87 (71.9)		
Sleeping hours				
< 8 h/day	140 (53.6)	64 (52.9)	0.019	0.892
≥ 8 h/day	121 (46.4)	57 (47.1)		
Smoking				
Yes	21 (8.0)	12 (9.9)	0.367	0.545
No	240 (92)	109 (90.1)		
Having breakfast^b^				
Always	66 (25.3)	27 (22.3)	.404	0.817
Sometimes	142 (54.4)	68 (56.2)		
Rarely	53 (20.3)	26 (21.5)		
Chewing process				
Eating slowly	36 (13.8)	18 (14.9)	2.146	0.342
Normal	170 (65.1)	70 (57.9)		
Eating fast	55 (21.1)	33 (27.3)		
Drink fluid with meal				
Yes	136 (52.1)	57 (47.1)	0.827	0.363
No	125 (47.9)	64 (52.9)		

^a^Significant

^b^Always = all the days in a week, Sometimes = 2–4 days/week, Rarely = less than 2 days/week

Table 3 shows no statistically significant difference regarding other health problems characteristics, or regarding emotional stress in the past 6 months.

**Table 3:** Comparison between IBS patients and normal students regarding health problems

	Normal *N* (%)	IBS patient *N* (%)	Chi-square	*p*
Chronic health problem				
Yes	34 (13)	20 (16.5)	0.835	0.361
No	227 (87)	101 (83.5)		
Medication use				
Yes	65 (24.9)	41 (33.9)	3.325	0.068
No	196 (75.1)	80 (66.1)		
Traveler’s diarrhea				
Yes	18 (6.9)	13 (10.7)	1.641	0.2
No	243 (93.1)	108 (89.3)		
Food allergy				
Yes	38 (14.6)	19 (15.7)	0.085	0.77
No	223 (85.4)	102 (84.3)		
Emotional stress in previous 6 months				
Yes	178 (70.4)	87 (75)	0.848	0.357
No	75 (29.6)	29 (25)		

Table 4 shows no statistically significant differences regarding food frequency questionnaire items.

**Table 4:** Comparison between IBS patients and normal students regarding food frequency questionnaire

	Normal *N* (%)	IBS patient *N* (%)	Chi-square	*p*
Vegetables				
Twice/week or less	90 (34.5)	37 (30.6)	0.568	0.451
3 times/week or more	171 (65.5)	84 (69.4)		
Fruits				
Twice/week or less	81 (31)	48 (40)	2.951	0.086
3 times/week or more	180 (69)	72 (60)		
Eggs, Milk and dairy products				
Twice /week or less	92 (35.2)	50 (41.3)	1.306	0.253
3 times/week or more	169 (64.8)	71 (58.7)		
Fish				
Twice/week or less	227 (87.3)	112 (92.6)	2.324	0.127
3 times/week or more	33 (127)	9 (7.4)		
Nuts				
Twice/week or less	225 (87.2)	106 (88.3)	0.095	0.758
3 times/week or more	33 (12.8)	14 (11.7)		
High-fat diet				
Twice/week or less	174 (66.9)	89 (73.6)	1.698	0.193
3 times/week or more	86 (33.1)	32 (26.4)		
Fast food				
Twice/week or less	153 (58.8)	74 (61.7)	0.272	0.602
3 times/week or more	107 (41.2)	46 (38.3)		
Home-made food				
Twice/week or less	74 (28.4)	43 (36.1)	2.323	0.127
3 times/week or more	187 (71.6)	76 (63.9)		
Coffee				
Twice/week or less	123 (47.3)	61 (50.4)	0.319	0.572
3 times/week or more	137 (52.7)	60 (49.6)		
Tea				
Twice/week or less	99 (38.1)	43 (35.8)	0.177	0.674
3 times/week or more	161 (61.9)	77 (64.2)		
Spicy food				
Twice/week or less	171 (65.5)	76 (62.8)	0.265	0.607
3 times/week or more	90 (34.5)	45 (37.2)		
Artificial sweeteners				
Twice/week or less	217 (83.8)	109 (90.1)	2.684	0.101
3 times/week or more	42 (16.2)	12 (9.9)		
Processed food				
Twice/week or less	141 (54.4)	69 (57.5)	0.311	0.577
3 times/week or more	118 (45.6)	51 (42.5)		

Table 5 shows a significant difference between IBS cases and normal participants regarding the presence of morbid anxiety (*p* = 0.001) and occurrence of traumatic events during childhood (*p* = 0.004). Causes of traumatic events were mentioned as follows: death of a close family member (46.6%), experiencing serious illness or major surgery (22%), extreme financial difficulty (15.7%), divorced parents (8.5%), and 7.2% experienced a natural disaster which affected their lives negatively.

**Table 5:** Comparison between IBS and normal students as regards depression and anxiety scores

	Normal *N* (%)	IBS patient *N* (%)	Chi-square	*p*
Anxiety				
Normal	120 (46)	32 (26.4)	13.18	0.001^a^
Borderline	63 (24.1)	39 (32.2)		
Abnormal	78 (29.9)	50 (41.3)		
Depression				
Normal	122 (46.7)	50 (41.3)	2.07	.355
Borderline	75 (28.7)	33 (27.3)		
Abnormal	64 (24.6)	38 (31.4)		
Traumatic events during childhood				
Yes	140 (53.6)	84 (69.4)	8.49	0.004*
No	121 (46.4)	37 (30.6)		

^a^Significant

Table 6 shows a statistically significant difference regarding morbid anxiety and depression in IBS subtypes (*p* = 0.02 and < 0.0001 respectively).

**Table 6:** Comparison between IBS subtypes as regards depression and anxiety scores

	IBS-D	IBS-C	Chi square	p
Anxiety				
Normal	8 (18.6)	24 (30.7)	7.78	0.02^a^
Borderline	10 (23.3)	29 (37.2)		
Abnormal	25 (58.1)	25 (32.1)		
Depression				
Normal	13 (30.2)	37 (47.4)	15.3	< 0.0001^a^
Borderline	7 (16.3)	26 (33.3)		
Abnormal	23 (53.5)	15 (19.3)		

^a^Significant

A multivariate logistic regression shows that IBS is significantly associated with positive family history, being a female, occurrence of traumatic events in childhood and performing regular exercise which proved to be protective from IBS (*p* values are < 0.0001, 0.003, 0.005, and 0.041 respectively) Table 7.

**Table 7:** Multivariate logistic regression to explore IBS-associated determinants

	B	S.E.	P value	Odds ratio	95% C.I. for odds ratio	
					Lower	Upper
Positive family history	1.934	0.267	< 0.0001^a^	6.917	4.096	11.681
Sex (Ref: male)	0.808	0.273	0.003^a^	2.243	1.315	3.828
Traumatic events	0.782	0.280	0.005^a^	2.187	1.264	3.783
Regular exercise	− 0.570	0.279	0.041^a^	0.565	0.327	0.977
Anxiety	0.390	0.284	0.169	1.477	0.847	2.576
Academic stage (Ref: Junior years)	0.299	0.265	0.258	1.349	0.803	2.266
Constant	− 2.633	0.384	0.000	0.072		

^a^Significant

## Discussion

Irritable bowel syndrome (IBS) is a gastrointestinal disorder, characterized by altered bowel habits with abdominal discomfort or pain with the absence of organic pathology. IBS is a highly prevalent disorder that has a great impact on patients’ quality of life [21]. In this work, we aimed to study the frequency of IBS in the selected study sample and to find out the associated factors related to this illness.

The frequency of IBS among studied medical students (no. = 382) was 31.7%. Moreover, 26.6% was IBS-D subtype and 37.4% was IBS-C subtype. This result is similar to a study performed in Saudi Arabia, King Abdulaziz University in Jeddah. They examined 597 medical students and interns and reported an IBS prevalence of about 31.8% [15]. Similarly, a medical school in Korea reported that 29.2% of medical students suffered from IBS [22]. In a medical school in Karachi, Pakistan, a study reported the prevalence of IBS to be 28.3% among its students [23]. Similarly, a study from Aga-Khan University in Pakistan found that 26% of its medical students suffer with IBS [24]. Other studies have reported a lower prevalence of IBS among medical students. In a Malaysian medical school, a study conducted on its students reported that 15.8% of them experienced IBS [25]. Two studies from Iran, the first from Shiraz University of Medical Sciences, the second from Gilan University, showed that 16.4% and 12.6% of medical students had IBS, respectively [9]. The highest prevalence of IBS among medical students was reported in a study from Japan, which revealed a prevalence of 35.5% among participants [13], while the lowest prevalence of IBS among medical students was reported in Northern China to be 9.3% [26]. This variation in the prevalence of IBS among medical students might be explained by either students not identifying symptoms as IBS-related or by variation in the prevalence of IBS in the general population.

The majority of IBS cases, in this work, were females with a significant difference (*p* = 0.006). A Korean study found that the prevalence of IBS was more in males than females, being 41% and 25%, respectively [22]. Another study in Pakistan stated that IBS was more common in males than females [24]. However, Wells et al. reported that female-to-male ratio in medical students affected with IBS was 2:1 [27]. In a Pakistani study, females had a significant increase in prevalence of IBS, compared to males, and a Malaysian study reported a similar result [23, 25]. Similarly, another study in Japan reported 41.5% of females had IBS symptoms compared to 13.8% of males [13]. An Indian study reported the same findings, being more in females [28]. Qureshi et al. reported that there is no exact effect of gender on the prevalence of the disease among medical students [21]. Studies reporting a higher prevalence of IBS among female students explained this gender predilection of the disease by possible variation in social characters and health-seeking behaviors. Also, the stress and symptoms encountered during the menstrual cycle might lead to more females reporting IBS-related symptoms. This was reported by Triadafilopoulos et al. who studied the prevalence IBS in women going through their climacteric and postmenopausal periods, including 170 postmenopausal and 58 premenopausal females [29]. On the opposite hand, studies reporting a higher prevalence of the disease among males suggested that the cultural barrier can be a limiting factor leading to less female students reporting the disease [24].

The mean age in this study was 20.69 ± 3.99. Some studies reported that it is more common among age groups below 25 years [30]. However, typical medical students’ age range does not vary significantly in different medical schools, which devalues the comparison based on this factor. A study from Jeddah, Saudi Arabia showed that the prevalence of IBS was higher in senior years, which was attributed to the higher study load rather than the difference in age [15]. This study revealed a significant difference regarding the academic year, being 52.8% in junior years (1st, 2nd, and 3rd years), and 47.2% in senior years. This difference may be related to the stress facing the new attendants to the faculty.

Physical and psychological stresses are considered major contributing factors to IBS etiology. The exact mechanism is not clear, but it is believed that the changes in central nervous system (CNS) in response to psychological and physical stressors lead to colonic spasms, which results in the manifestation of IBS symptoms [3]. Medical students are likely to be under a lot of stress due to the tremendous academic load [24]. In this study, we assessed psychological problems and found that almost two-thirds of our students with IBS were having emotional stress in their past 6 months, 32.9% were anxious and 26.1% were depressed, 26.3% were borderline anxious and 27.2% were borderline depressed. The current results showed a significant difference regarding anxiety, being higher in IBS patients, especially the diarrhea-predominant subtype (IBS-D). Depression showed highly significant difference in relation to IBS subtypes, being higher in the diarrhea predominant subtype too. Similarly, a study in Jeddah, Saudi Arabia, reported that 40.1% of students with IBS had morbid anxiety, and 41.9% had morbid depression, compared to IBS students with borderline depression (29.5%) and with no depression (31.5%) [15]. Similar results were also obtained from King Saud University in Saudi Arabia and from Malaysian studies [25, 31]. A Pakistani study showed that 55.8% of IBS causes were associated with stress [23]. The Malaysian study also reported higher rates of depression in IBS students [25]. Okami et al. in Japan also reported a significantly higher level of anxiety and depression among IBS students [13].

Regarding special habits, unexpectedly, smoking was not found to be associated with high IBS prevalence and only 10% of IBS students were smokers. A study from Jeddah, Saudi Arabia showed similar results with no significant association between smoking and having IBS [15]. However, a study from India reported an association between cigarettes smoking and IBS [28]. Regular exercise as a lifestyle habit was significantly protective against developing IBS in this study and few other studies. Further, 71.9% of students with IBS were not performing regular exercise in this study. A study from Saudi Arabia found that IBS prevalence was higher (37.3%) among students who did not exercise regularly compared to those who did (26.1%) [15]. Similarly, a study in Japan reported that students with IBS were performing less exercise than students with no IBS [13].

Eating habits and dietary balance can play a very important role in the development and severity of IBS-related symptoms. These factors are particularly important in students as they are more likely to be less cautious with their diet. Items discussed were chewing well; having breakfast; drinking plenty of fluids; favoring fresh food like fruits, vegetables, nuts, fish, and dairy products; consuming excess spicy or processed food; and drinking excess coffee or tea. Unfortunately, we did not find significant relation between types of food or food habits and IBS prevalence. Similar findings were reported in the study from Jeddah, Saudi Arabia that showed no relation between different food types and the prevalence of IBS [15]. Also, the Malaysian study reported no relation between consumption of chilly or high fiber diet and the risk of developing IBS [25]. On the contrary, the study at King Saud University, Saudi Arabia found that 15.5% of IBS symptoms can be related to dietary factors [31]. A Japanese study showed that patients with IBS consumed more processed food than fresh food [13]. An Indian study reported that fatty food increased the risk of having IBS [28]. Other studies reported that spicy and salty foods were related to development of IBS [22, 32]. Discussing food allergy as a separate entity, no relation was found in this study between food allergy and IBS development. This finding was in line with Almutairi et al. who reported no relation between food allergy and probability of IBS [33]. Unlike that was reported by Carroccio et al., who reported that IBS was more prevalent in those who were allergic to certain types of food [34].

In this study, no significant association was found between travelers’ diarrhea and IBS. Almutairi et al. reported that travelers’ diarrhea was associated with an increase in the probability of IBS [33].

### Limitations of the study

This study was cross-sectional in design and accordingly causation cannot be proved. A prospective study would be needed to confirm causation. Also, the data used in this study was collected by self-administered questionnaires which is subjective and accordingly can introduce some bias.

## Conclusion and recommendations

Medical students are likely to be subjected to substantial amounts of stress, anxiety, and depression, those major factors that can account for the increased prevalence of IBS, along with other factors. More studies are probably needed to evaluate the exact impact of IBS on the students’ quality of life. Raising awareness among students regarding IBS-related symptoms and factors leading to its development would probably play an important role in mitigating the impact of the disease on their quality of life. Meanwhile, reducing risk factors and implementing preventive strategies are important in controlling the disease and decreasing its undesirable effect.

## Data Availability

The datasets used and/or analyzed during the current study are available from the corresponding author on reasonable request.
